# Position Estimation and Local Mapping Using Omnidirectional Images and Global Appearance Descriptors

**DOI:** 10.3390/s151026368

**Published:** 2015-10-16

**Authors:** Yerai Berenguer, Luis Payá, Mónica Ballesta, Oscar Reinoso

**Affiliations:** Departamento de Ingeniería de Sistemas y Automática, Miguel Hernández University, Avda. de la Universidad s/n, Elche (Alicante) 03202, Spain; E-Mails: lpaya@umh.es (L.P.); m.ballesta@umh.es (M.B.); o.reinoso@umh.es (O.R.)

**Keywords:** computer vision, position estimation, mapping, omnidirectional images, global appearance, Radon transform

## Abstract

This work presents some methods to create local maps and to estimate the position of a mobile robot, using the global appearance of omnidirectional images. We use a robot that carries an omnidirectional vision system on it. Every omnidirectional image acquired by the robot is described only with one global appearance descriptor, based on the Radon transform. In the work presented in this paper, two different possibilities have been considered. In the first one, we assume the existence of a map previously built composed of omnidirectional images that have been captured from previously-known positions. The purpose in this case consists of estimating the nearest position of the map to the current position of the robot, making use of the visual information acquired by the robot from its current (unknown) position. In the second one, we assume that we have a model of the environment composed of omnidirectional images, but with no information about the location of where the images were acquired. The purpose in this case consists of building a local map and estimating the position of the robot within this map. Both methods are tested with different databases (including virtual and real images) taking into consideration the changes of the position of different objects in the environment, different lighting conditions and occlusions. The results show the effectiveness and the robustness of both methods.

## 1. Introduction

Nowadays, there are countless kinds of robots with innumerable configurations. Among them, mobile robots have been extended in recent years due to their flexibility, as they are able to change their position during operation. Usually, these robots have to solve a task in an unknown environment where the robot must estimate its position to be able to arrive at the target point, avoiding obstacles. There are two main approaches to solve the localization problem. First, the robot may have a map of the environment. In this case, the robot has to calculate its position within the map. Second, the robot may not have any *a priori* knowledge of the environment; thus, it has to create the map and then calculate its position. If these processes are carried out simultaneously, the problem is known as SLAM (simultaneous localization and mapping).

The robot can be equipped with many kinds of sensors to solve these problems, such as lasers, cameras, *etc*. They provide the robot with environmental information in different ways (e.g., lasers measure the distance to the nearest objects around the robot). This information is processed by the robot and permits building a map and estimating its position and orientation. With these data, the robot must be able to carry out its work autonomously.

Along the last few years, much research has been developed on robot mapping and localization using different kinds of sensors, and many algorithms have been proposed to solve these problems. Many these works use visual sensors, since they permit many possible configurations, and they provide the robot with very rich information from the environment. In this work, we consider a robot equipped with an omnidirectional vision system [1]. We can find many previous works that use omnidirectional images in mapping and localization tasks, such as [2,3,4,5]. Valiente *et al*. [2] present a comparison between two different visual SLAM methods using omnidirectional images. Maohai *et al*. [3] propose a topological navigation system using omnidirectional vision. Garcia *et al.* [4] present a map refinement framework that uses visual information to refine the topology of the environment. At last, Garcia *et al.* [5] make a survey of vision-based topological mapping and localization methods.

Traditionally, the developments in mobile robots using visual sensors are based on the extraction and description of some landmarks from the scenes, such as SIFT (scale-invariant feature transform) [6] and SURF (speeded-up robust features) [7] descriptors. This approach presents some disadvantages: the computational time to calculate and compare the descriptors is usually very high; thus, these descriptors may not be used in real time, and this leads to relatively complex mapping and localization algorithms. As an advantage, only a few positions need to be stored in the map to make the localization process possible.

More recently, some works proposed using the global information of the images to create the descriptors. These techniques have been demonstrated to be a good option to solve the localization and navigation problems in 2D. Chang *et al.* [8], Payá *et al.* [9] and Wu *et al.* [10] propose three examples of this approach. In [11], several methods to obtain global descriptors from panoramic scenes are analyzed and compared to prove their validity in map building and localization. The majority of these global appearance descriptors can be used in real time, because the computational time to calculate and handle them is low, and they usually lead to more straightforward mapping and localization algorithms.

In this work, we propose a solution to the mapping and localization problems using only the visual information captured by an omnidirectional vision system mounted on the robot. This system is composed of a camera pointing to a hyperbolic mirror and captures omnidirectional images of the environment. Each scene is described with a single global-appearance descriptor.

Our starting point is a database of omnidirectional images captured on a grid of points in the environment where the robot has to navigate.

Compared to previous works, the contributions of this paper are:

1. Describing the global appearance of a set of images: We develop a method based on the Radon transform [12] and the gist descriptor [13] to create a visual model of the environment. We have not found any previous work that uses the Radon transform as a global appearance descriptor to solve the mapping and localization problems in robotics.
2. Solving the mapping and localization problems with these descriptors: This contribution is two-fold:
  (a) Solving the localization problem with accuracy, with respect to a visual model (map) previously created: In this model, the positions where the images were captured are known.
  (b) Solving the localization problem with respect to a visual model where the capture positions of the images are not known: We have implemented a local mapping algorithm, and subsequently, we solve the localization problem.

The experiments have been carried out with several different image databases. The first one has been created synthetically from a virtual room. We also use some real databases to test the validity of our method.

The remainder of this paper is structured as follows. Section 2 introduces the concept of global appearance and the descriptors that we have developed. Section 3 presents the algorithm that we have implemented to solve the local mapping problem (spring method). Section 4 describes our localization method to estimate the position of the robot. In Section 5, the experiments and results are presented. At last, a discussion is made in Section 6, and Section 7 outlines the conclusions.

## 2. Global Appearance of Omnidirectional Images

Methods based on the global appearance of the scenes constitute a robust alternative compared to methods based on landmark extraction. The key is that the global appearance descriptors represent the environment through high level features that can be interpreted and handled easily, and with a reasonably low computational cost.

This section presents the transform that we have employed to describe the scenes (omnidirectional images). Each scene is represented through only one descriptor that contains information of the global appearance without any segmentation or local landmark extraction. We also present the distance measures we use to compare descriptors, and we study their properties.

Any new global appearance description method should satisfy some properties: (1) it should make a compression effect in the image information; (2) there should be a correspondence between the distance between two descriptors and the metric distance between the two positions where the images were captured; (3) the computational cost to calculate and compare them should be low, so that the approach can be used in real time; (4) it should provide robustness against noise, changes in lighting conditions, occlusions and changes in the position of some objects in the environment; and, at last, (5) it should contain information of the orientation that the robot had when it captured the image.

The description method we have employed is mainly based on the Radon transform. We also make use of the gist descriptor, because of its properties in localization tasks [11].

### 2.1. Radon Transform

The Radon transform was initially described in [12]. It has been used traditionally in some computer vision tasks, such as shape description and segmentation ([14,15]).

The Radon transform in 2D consists of the integral of a 2D function over straight lines (line-integral projections). This transform is invertible. The inverse Radon transform reconstructs an image from its line-integral projections. For this reason, it was initially used in medical imaging (such as CAT scan and magnetic resonance imaging (MRI)).

The Radon transform of a 2D function f(i,j) can be defined mathematically as:

(1) $$R\left\{f\left(i,j\right)\right\}=\lambda_{f}\left(p,\text{ϕ}\right)={\;\int \int \;}_{-\infty }^{+\infty }f\left(i,j\right)\;\text{δ}\left(p-\vec{r}-\hat{\vec{p}}\right)\;\mathrm{di}\;\mathrm{dj}$$

where δ is the Dirac delta function (δ(x)=1 when x=0, and δ(x)=0 elsewhere). The integration line is specified by the radial vector $\vec{p}$ that is defined by $\vec{p}=\hat{\vec{p}}\cdot p$, where $\hat{\vec{p}}$ is a unitary vector in the direction of $\vec{p}$. *p* is the $\vec{p}$ magnitude:

(2) $$\begin{array}{cc} p\; & =|\vec{p}| \end{array}$$

The line-integral projections evaluated for each azimuth angle, ϕ, produce a 2D polar function, $\lambda_{f}$, that depends on the radial distance *p* and the azimuth angle ϕ. $\vec{r}$ is a cluster of points that is perpendicular to $\vec{p}$.

**Figure 1:**
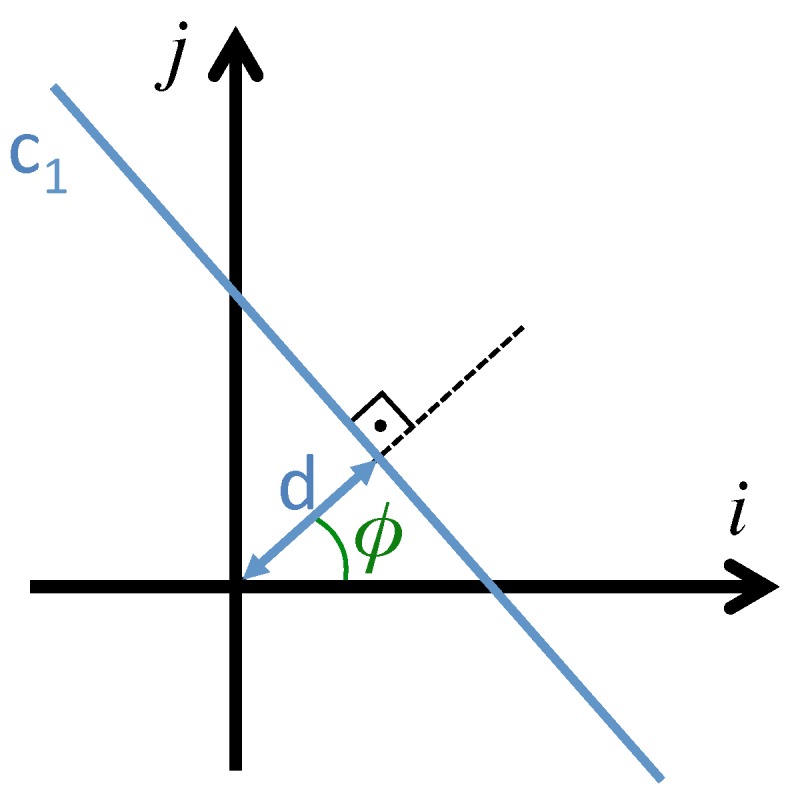
Line parametrization through the distance to the origin *d* and the angle between the normal line and the *i* axis, ϕ.

The Radon transform of an image im(i,j) along the line $c_{1}\left(d,\text{ϕ}\right)$ (Figure 1) can be expressed more clearly by the following equivalent expression:

(3) $$R\left\{im\left(i,j\right)\right\}=\int_{R}im\left(i^{\prime }\cos\text{ϕ}-j^{\prime }\sin\text{ϕ},i^{\prime }\sin\text{ϕ}+j^{\prime }\cos\text{ϕ}\right)\;\mathrm{ds}$$

where:

(4) $$\left[\;\begin{array}{c} i^{\prime }\\ j^{\prime } \end{array}\;\right]=\left[\;\begin{array}{cc} \cos\text{ϕ} & \sin\text{ϕ}\\ -\sin\text{ϕ} & \cos\text{ϕ} \end{array}\;\right]\cdot \left[\;\begin{array}{c} i\\ j \end{array}\;\right]$$

**Figure 2:**
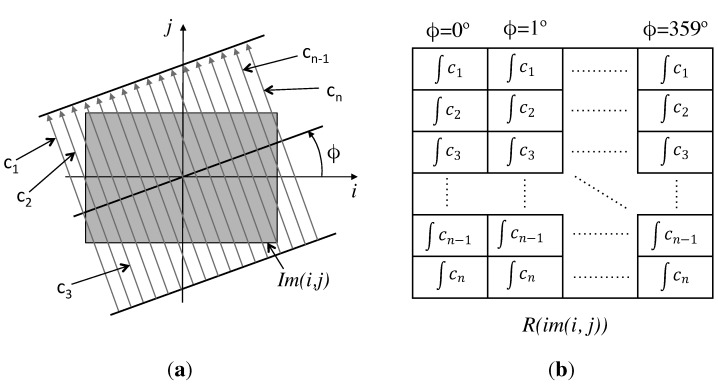
(**a**) Integration paths to calculate the Radon transform of the image im(i,j) in the ϕ direction; (**b**) Radon transform matrix of the image im(i,j).

When the Radon transform is applied to images, it calculates the image projections along the specified directions through a cluster of line integrals along parallel lines in these directions. The distance between the parallel lines is usually one pixel. Figure 2a shows the integration paths to calculate the Radon transform of an image in the ϕ direction, and Figure 2b shows the value of each component of the Radon transform in a simplified notation.

**Figure 3:**
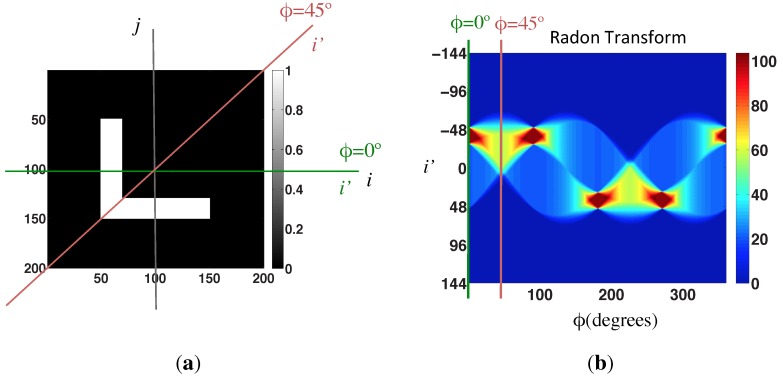
(**a**) Sample image; (**b**) Radon transform of the sample image.

Figure 3 shows a sample black and white image, on the left, and its Radon transform, on the right. Furthermore, it shows graphically the process to calculate the Radon transform.

#### 2.1.1. Radon Transform Properties

The Radon transform has several properties that make it useful in localization tasks using omnidirectional images. These properties are the following:

- Linearity: The Radon transform meets the linearity property as the integration operation is a linear function of the integrand:
  (5) R{αf+βg}=αR{f}+βR{g}
- Shift: The Radon transform is a variant operation to translation. A translation of the two-dimensional function by a vector $\vec{r_{0}}=\left(i_{0},j_{0}\right)$ has a translation effect on each projection. This translation is given by a distance $\vec{r}\cdot \left(\cos\text{ϕ},\sin\text{ϕ}\right)$.
- Rotation: If the image is rotated an angle ${\text{ϕ}}_{0}$, it implies a shift ${\text{ϕ}}_{0}$ of the Radon transform along the variable ϕ (columns shift).
- Scaling: A scaling of *f* by a factor *b* implies a scaling of the *d* coordinate and amplitude of the Radon transform by a factor *b*:
  (6) $$R\left\{f\left(\frac{i}{b},\frac{j}{b}\right)\right\}=\left|b\right|\lambda_{f}\left(\frac{d}{b},\text{ϕ}\right)$$

### 2.2. Gist Descriptor

The gist descriptors try to imitate the human perception system to describe the scenes. They identify regions with a prominent color or texture in relation to the environment. The gist concept was introduced by Oliva and Torralba [13] with the name holistic representation of the spatial envelope. The authors proved that this description method creates a multidimensional space in which the scenes with the same semantic category (e.g., streets, sky, buildings, *etc*.) are projected in near points.

Mathematically, the method tries to codify the spatial information by means of 2D Fourier transform in several regions of the image. These regions are distributed in a regularly-spaced grid. The set of descriptors in each region is dimensionally reduced by PCA (principal component analysis) to generate a unique scene descriptor with reduced dimension. In recent works, wavelet pyramids have been used instead of the Fourier transform, as in [16], where the authors carried out several experiments with different groups of natural images (coasts, forests, open fields, deserts, *etc.*) and artificial images (images of buildings, doors, interior of buildings, roads, *etc.*). The developed descriptors worked successfully to classify these images according to the above properties.

Recent works use the prominence concept together with gist, which highlights the zones that differ more from their neighbors [17]. This descriptor is built with the information of intensity, orientation and color.

**Figure 4:**
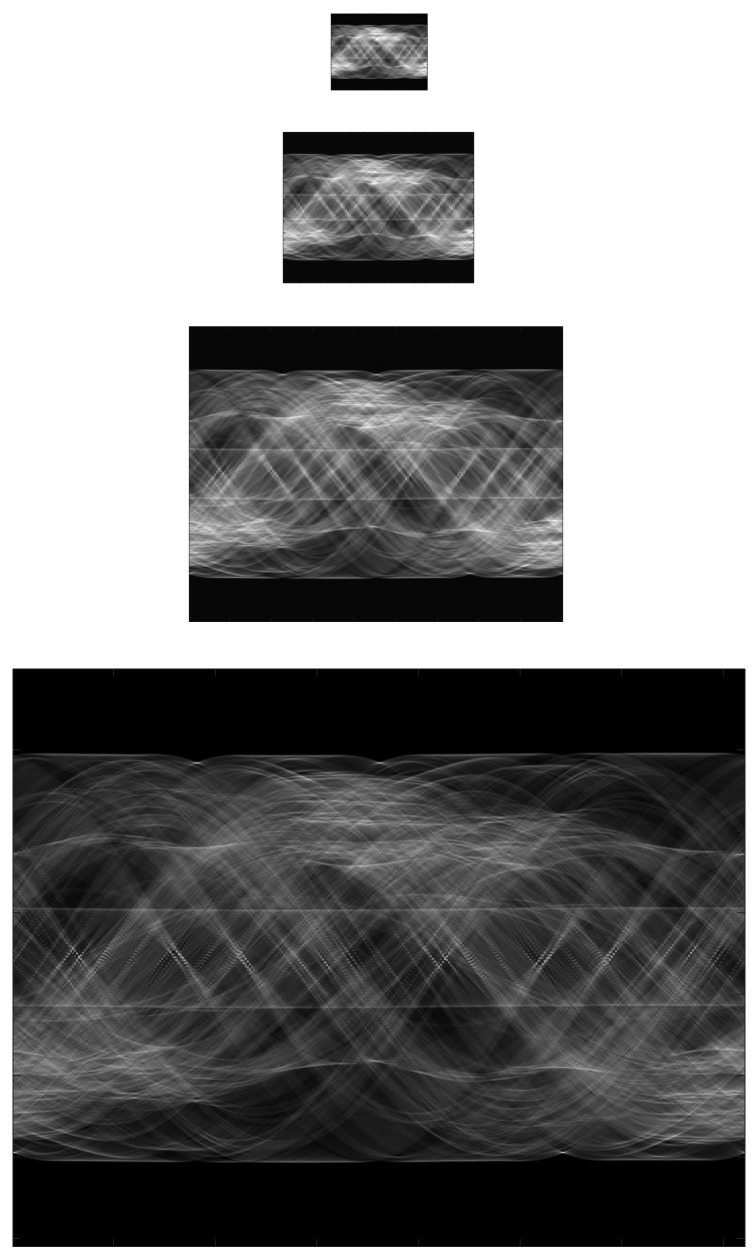
Example of a Radon transform four-level pyramid.

In this work, some changes are made to this descriptor, since it will be used for a different purpose. We work with a set of indoor images with many similarities between them. The descriptor must be able to work with these images in such a way that the distance in the descriptor space reflects the geometrical distance between the points where the images were captured. Additionally, the Radon transform has been used instead of images. Since this transform can be interpreted as a grayscale image, only the concept of prominence is used (but not color information). The steps we have implemented to build the gist descriptor are:

1. Building a pyramid of Radon transforms: At first, a Gaussian pyramid of *n* Radon transforms is created to describe the Radon transform features in several scales. The first level of the pyramid is the original Radon transform. Each new level is obtained from the previous one, applying a Gaussian low-pass filter and subsampling it to reduce its resolution. Figure 4 shows an example of a Radon transform four-level pyramid.

2. Gabor filtering: In order to incorporate the orientation information in the descriptor, each Radon transform of the two levels of the pyramid is filtered by four Gabor filters with different orientations $\theta ={\left\{0,45,90,135\right\}}^{\circ }$. As a result, four matrices per pyramid level are obtained, with orientation information in the four analyzed directions.
3. Blockification. Finally, a dimensionality reduction process is needed. With this aim, we group the pixels of every matrix in blocks calculating the average intensity value that has the pixels in each block. We have decided to use horizontal blocks. This type of block is interesting, because the information in each block is independent of the robot orientation. The final descriptor will be composed of *b* horizontal blocks per pyramid level and Gabor filter, since it will be used only for localization purposes (additionally, we could have defined vertical blocks if we had needed to compute the robot orientation [11]).

**Figure 5:**
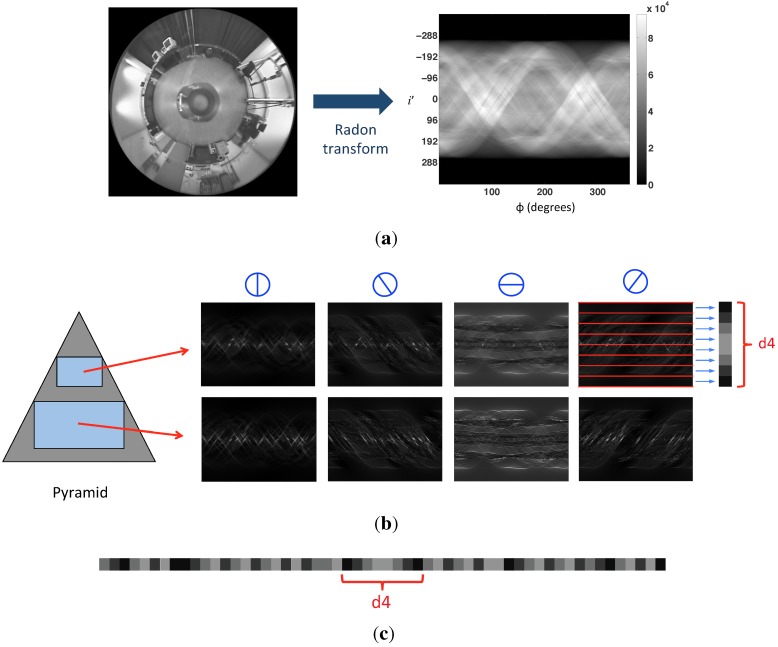
(**a**) The Radon transform of an omnidirectional image; (**b**) Matrices obtained after applying each of the four Gabor filters to the two levels of the pyramid; (**c**) Final composition of the gist descriptor.

Figure 5 shows the process to obtain the gist descriptor. In the Figure 5a, we can observe the Radon transform of an omnidirectional image. The use of each Gabor filter in the Radon transform and the blockification is shown in Figure 5b. In this case, n=2 levels, and b=8 horizontal blocks. Finally, in Figure 5c, the final composition of the gist descriptor is shown. The size of the descriptor is equal to 4·n·b components.

### 2.3. Phase-Only Correlation

In this subsection, we present the method employed to compare the Radon transform of two images.

In general, a function in the frequency domain is defined by its magnitude and its phase. Sometimes, only the magnitude is taken into account, and the phase information is usually discarded. However, when the magnitude and the phase features are examined in the Fourier domain, it follows that the phase features contain also important information, because they reflect the characteristics of patterns in the images.

Oppenheim and Lim [18] have demonstrated this by reconstructing images using the full information from the phase with unit magnitude. This shows that the images resemble the originals, in contrast to reconstructing images using the full information from the magnitude with uniform phase.

Phase-only correlation (POC), proposed in [19], is an operation made in the frequency domain that provides a correlation coefficient between two images [20]. In our case, the objective is to compare the Radon transforms of two different images. This does not affect the POC performance, because the Radon transform can be interpreted as an image. Therefore, we explain POC using images.

The correlation between two images $im_{1}\left(i,j\right)$ and $im_{2}\left(i,j\right)$ calculated by POC is given by the following equation:

(7) $$C\left(i,j\right)=F^{-1}\left\{\frac{{\mathrm{IM}}_{1}\left(u,v\right)\cdot {\mathrm{IM}}_{2}^{*}\left(u,v\right)}{\left|{\mathrm{IM}}_{1}\left(u,v\right)\cdot {\mathrm{IM}}_{2}^{*}\left(u,v\right)\right|}\right\}$$

where ${IM}_{1}$ is the Fourier transform of Image 1 and ${IM}_{2}^{*}$ is the conjugate of the Fourier transform of Image 2. $F^{-1}$ is the inverse Fourier transform operator.

To measure the similitude between the two images, the following coefficient is used:

(8) $$sim_{POC}\left(im_{1},im_{2}\right)=max\{\left(C\left(i,j\right)\right\}$$

This coefficient takes values in the interval [0,1], and it is invariant under shifts in the *i* and *j* axes of the images. Furthermore, it is possible to estimate these shifts $\Delta_{i}$ and $\Delta_{j}$ along both axes by:

(9) $$\left(\Delta_{i},\Delta_{j}\right)={argmax}_{\left(i,j\right)}\left\{C\left(i,j\right)\right\}$$

If we use Radon transforms instead of images, the value $\Delta_{i}$ allows us to estimate the change of the robot orientation when capturing the two images.

This way, POC is able to compare two images independently on the orientation, and it is also able to estimate this change in orientation.

### 2.4. Distance Measure

To carry out the localization process, we need any mechanism to compare descriptors (distance measure). We have tested two different methods.

First, we can measure the distance between the Radon transform (RT) of two scenes by means of the POC magnitude (POC distance) by the following equation:

(10) $$dist_{POC}\left(RT_{1},RT_{2}\right)=1-sim_{POC}\left(RT_{1},RT_{2}\right)$$

Second, we can make use of the gist descriptor to obtain a distance measure between images following the next steps:

1. Firstly, the two omnidirectional images are transformed by the Radon transform to create two descriptors $RT_{1}$ and $RT_{2}$.
2. Secondly, $RT_{1}$ and $RT_{2}$ are transformed with the gist approach (Section 2.2) to obtain $g_{1}$ and $g_{2}$.
3. Finally, the Euclidean distance between $g_{1}$ and $g_{2}$ is obtained (gist distance).

**Figure 6:**
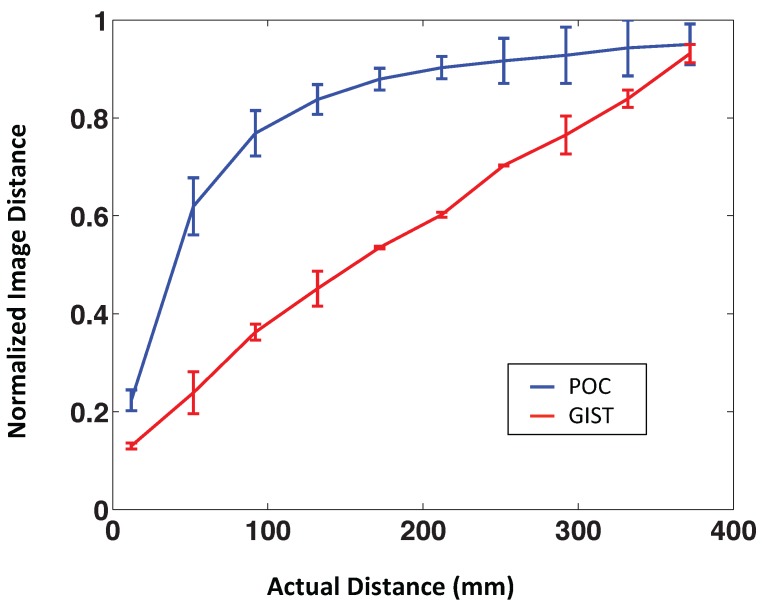
POC distance and gist distance *vs*. geometric distance. Each distance measure has been calculated along four different paths.

**Figure 7:**
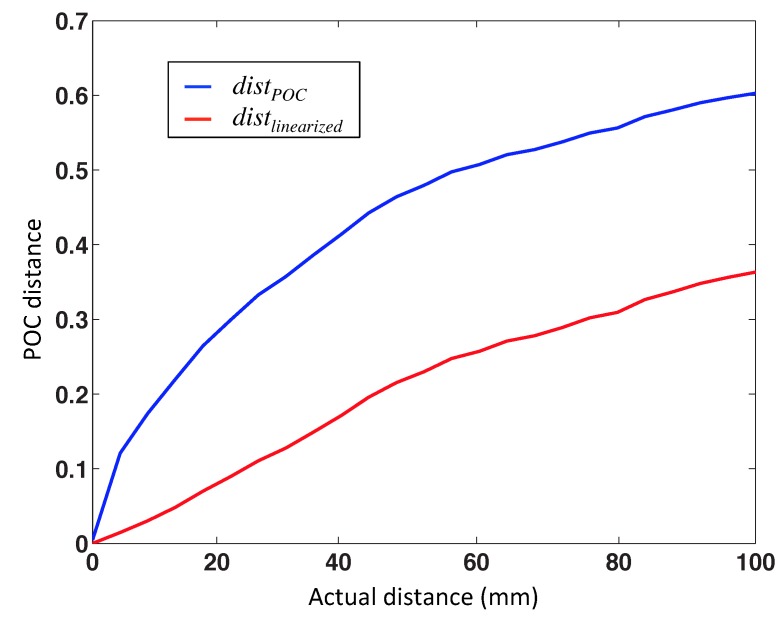
Phase-only correlation (POC) distance and POC distance linearized *vs*. geometric distance.

Figure 6 shows the POC and the gist distance *vs.* the geometric distance between the points where the images were captured. According to this figure, the POC distance shows a nonlinear behavior. This method seems to be a promising option to identify the nearest image, but it is not good to estimate the distance between images. The POC distance can be linearized by the following expression:

(11) $$dist_{linearized}=dist_{POC}^{2}$$

where $dist_{linearized}$ is the final distance and $dist_{POC}$ the original POC distance. Figure 7 shows the POC distance and the linearized POC distance *vs*. the geometric distance of one aleatory linear path using our virtual database.

On the other hand, Figure 6 shows that the gist distance presents a quite linear behavior, so we expect it to be useful in mapping tasks.

## 3. Local Map Building (Spring Method)

In this section, we address the problem of building local maps of an unknown environment. We start from a set of omnidirectional images captured from different points of the environment to map, and we have no information of the capture positions nor of the order they were captured. Our objective is to estimate these positions in local regions of the environment. Thanks to this, we will be able to carry out a subsequent localization process (Section 4).

The mapping problem is solved using a mass-spring model, which represents the final map as a graph formed by nodes linked through connectors. Each node contains one image, and the connectors represent the neighbor relationships between two nodes, so that the images that have been captured sequentially are expected to belong to nodes that are connected together.

The only information we use to build the map is the distance among image descriptors. Once the map is built, we make use of the Procrustes transformation [21] to compare the resulting map with the actual map.

**Figure 8:**
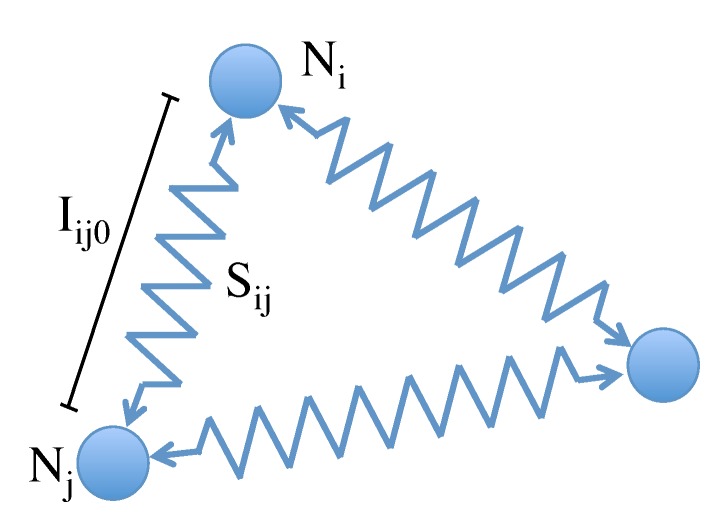
Spring model.

The method is based on a physical system of forces named the spring model [22,23]. The model is formed by the combination of two principles: Hooke’s law and Newton’s second law. Figure 8 shows the physical model scheme using three nodes. Each node $N_{i}$ corresponds to an image, and they are linked with the springs $S_{ij}$ whose natural length $l_{ij0}$ is the distance between descriptors of images *i* and *j*. This method connects all of the nodes that have been previously chosen to create the local map and estimate the accuracy of the process.

This method is based on the calculation of the elastic forces exerted by the springs on each node. Once the resulting force exerted on each node has been calculated, we proceed to obtain the acceleration, speed and position of each node.

The mapping process consists of solving at each time instant the next equations:

(12) $$\vec{F_{i}}=\sum_{S_{ij}\in S}\left(-K_{ij}\left(l_{ij0}-l_{ij}\right)-b_{ij}\left(v_{i}-v_{j}\right)\right)$$

(13) $$\vec{a_{i}}=\frac{\vec{F_{i}}}{m_{i}}$$

(14) $$\vec{v_{i}}\left(t+\Delta T\right)=\vec{v_{i}}\left(t\right)+\vec{a_{i}}\left(t\right)\cdot \Delta T$$

(15) $$\vec{r_{i}}\left(t+\Delta T\right)=\vec{r_{i}}\left(t\right)+\vec{v_{i}}\left(t\right)\cdot \Delta T,$$

where Equation (12) corresponds to Hooke’s harmonic oscillator law. The resulting force on node *i*, $\vec{F_{i}}$, depends on the length of the springs $l_{ij}$ and the elastic constant of the spring $K_{ij}$. A damper with damping constant $b_{ij}$ has also been added between nodes, as it helps to improve the convergence of the algorithm. Newton’s second law allows us to obtain the acceleration $\vec{a_{i}}$ using the node mass $m_{i}$. To simplify the system of forces, we used a mass equal to one for all nodes. Finally, the last two expressions are used to update the calculation of the speed $\vec{v_{i}}$ and the position $\vec{r_{i}}$ of all nodes in the system at each iteration (for each time increment, ΔT).

## 4. Localization Methods

In this section, we address the localization problem. Initially, the robot has a map of the environment. It is composed of a set of omnidirectional images of this environment. Then, the robot captures an image from an unknown position (test image). Comparing this image to the visual information stored in the map, the robot must be able to estimate its position.

To develop the algorithms, we take into consideration two different situations: (1) the positions where the map images were captured are known (pure localization); and (2) these positions are not known, and they must be estimated prior to the localization process (local map building and localization).

We have designed these algorithms to solve the problems:

1. Detecting the nearest image of the map.
2. Local mapping and localization.

The first method allows us to know the nearest image of the map (the most similar to the test image). Thanks to this information, we know that the robot is located in the surroundings of the corresponding image. The second method consists of calculating the image distance between the nearest images of the map and the test image. The number of nearest images is a parameter that can be modified to optimize the method. With these distances, we use the spring method to estimate the position of each image. As a result, we have the local map and the localization of the robot. These two methods are detailed in the following subsections.

### 4.1. Nearest Position of the Map

The operation consists of the following steps:

1. The robot captures an omnidirectional image from its current unknown position (test image). The objective is to estimate this position.
2. This image is transformed using the Radon transform.
3. The Radon transform of the test image is compared to all of the Radon transforms of the map using the POC comparison. As a result, we know which is the most similar image in the map to the test image.
4. The position where this omnidirectional image was taken is the nearest neighbor.

After this process, we assume that the robot is around this position. This is an absolute localization process, since we did not know the previous position of the robot nor its path. The accuracy depends mainly on the distance between the images of the map.

### 4.2. Local Mapping and Localization

In this subsection, we propose a method to localize the robot without any information of the position where the images of the map were captured. We have the Radon transforms of each image, but we do not have information about the position in which these images were captured. This localization method consists of the following steps:

1. We choose a number of nearest neighbors to use. These nearest neighbors are the descriptors that present the lowest POC distance to the Radon transform of the test image.
2. The gist descriptor of each Radon transform is calculated including the test image.
3. The distance between each pair of gist descriptors is obtained. We test two different distance measures: the Euclidean and the POC distance.
4. The spring method is used with these distances. This step provides a relative position between each gist descriptor. As a result, the position of the map images (local map) and the position of the test image are now known. This is our estimation of the robot position.
5. To estimate the localization error with respect to the local map, we must take into account that this map may present a rotation and a scale factor comparing to the actual position of the images. To consider these effects, we use a Procrustes approach [21] to estimate the error. This method removes the rotation and scale factor of the local map. The final error is the distance between the actual position of the robot and the calculated position after removing both effects.

**Figure 9:**
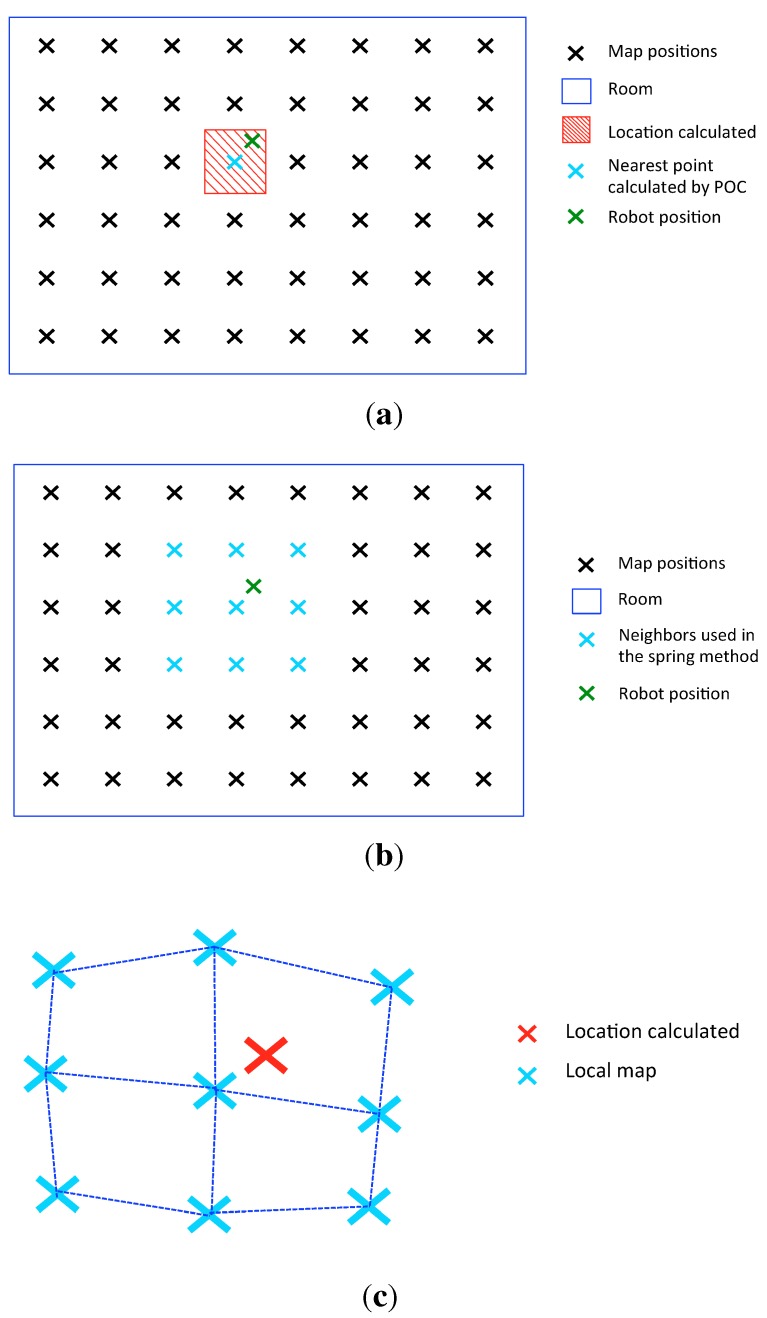
(**a**) Nearest position of the map; (**b**) Map positions and neighbors used in the spring method; (**c**) Local mapping and localization.

Figure 9a shows the location area calculated by the first method (nearest position of the map). Figure 9b shows the location calculated by the second method with the nine nearest neighbors (local mapping and localization). In this figure, the map positions are represented at the actual capture points for representation purposes, but the algorithm does not know these positions. In Figure 9c, the local map created and the localization of the robot is shown. To make this local map, the initial position of each node to launch the spring method is random. This randomization may have an effect on the final result. For this reason, we run the algorithm several times. The results of this spring method are the local map and the robot localization.

## 5. Experiments and Results

In this section, first, we present the virtual database created to test the methods and the results obtained with it. Second, we describe the database of real images that we have used and the results obtained with this second database.

### 5.1. Virtual Database

**Figure 10:**
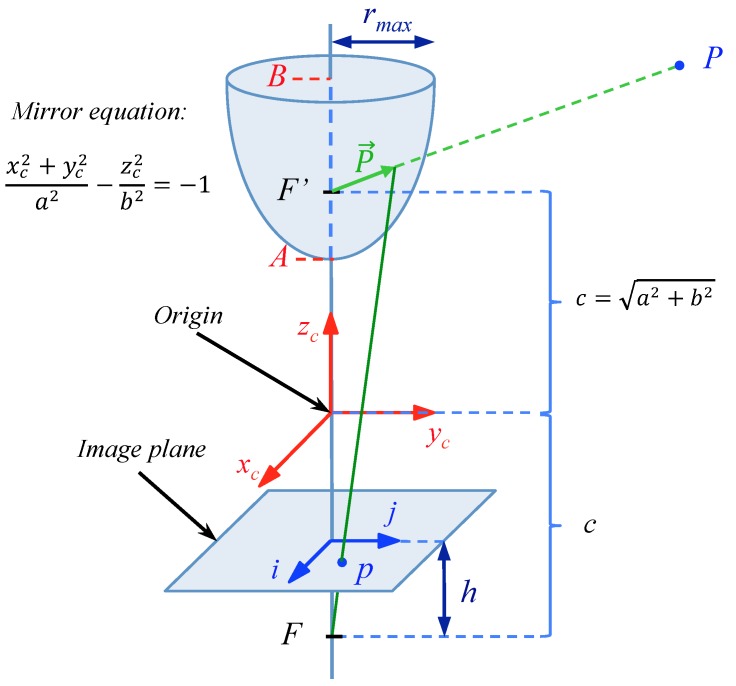
Catadioptric system used to capture the synthetic omnidirectional images.

**Figure 11:**
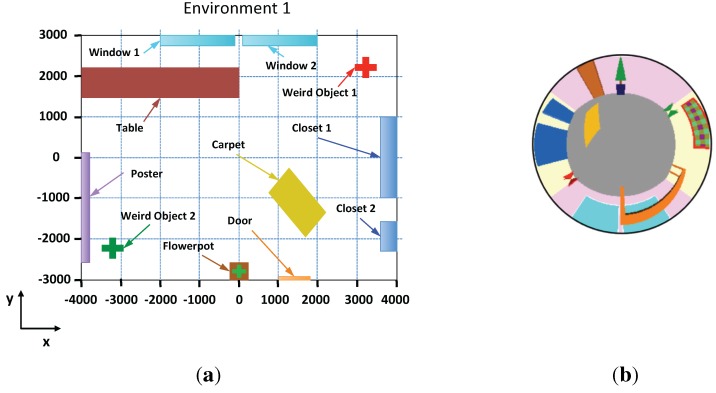
(**a**) Bird eye’s view of the virtual environment; (**b**) Example of an omnidirectional image captured from the points x = 0 and y = 0.

In order to check the performance of the proposed technique, we have created a virtual environment that represents an indoor room. In this environment, it is possible to create omnidirectional images from any position using the catadioptric system showed in Figure 10. Figure 11a shows a bird’s eye view of the environment.

The omnidirectional images have 250 × 250 pixels, and they have been created using a catadioptric system composed of a camera and a hyperbolic mirror whose geometry is described in Figure 10. The parameters used in the mirror equation are a=40 and b=160.

Several images have been captured in the environment to create the map from several positions on the floor. The map is composed of 4800 images captured on a 8 × 6 meter grid with a step of 10 cm between images. To carry out the experiments, we can change the step of the grid to test its influence. We must take into account that the higher the grid step, the fewer images compose the map. Figure 11b shows one sample omnidirectional image of the environment created with our program.

### 5.2. Results Obtained with the Virtual Database

To test our methods, we will study the influence of some parameters, such as the grid step of the map and the number of neighbors used in the spring method. In this subsection, we compare the results of the tests to try to optimize each method.

Firstly, we analyze the first method (obtaining the nearest position of the map) and the influence of the grid step of the map. Secondly, we analyze the second method (local mapping and localization) and the influence of the grid step of the map and the number of nearest neighbors.

#### 5.2.1. First Method: Detecting the Nearest Image of the Map

In this experiment, we analyze four different step sizes between consecutive map positions. The distances that we will use are 100, 200, 300 and 400 mm. The size of the grid is 8 m × 6 m in all cases, so the number of map images depends on the step size. It will have an important influence on the computational cost.

**Figure 12:**
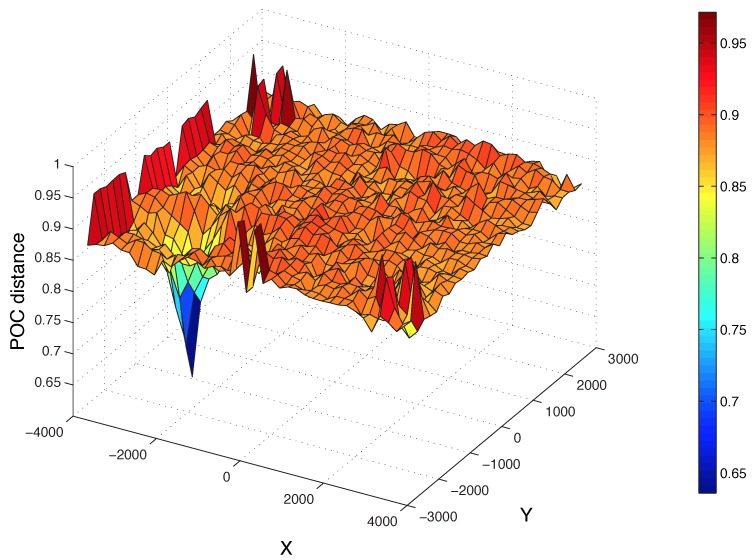
Distance between the Radon transform of a sample test image and all of the images of the map. The position of the test image is x = −2239 mm and y = −1653 mm.

Figure 12 shows the POC distance (Equation (10)) between the Radon transform of a test image and each image of the map (200 × 200). The position of the test image is x = −2239 mm and y = −1653 mm. Additionally, the corresponding position according to this figure (the minimum of the 2D function) is x = −2200 and y = −1600. As we can see, the distance decreases sharply around this position.

**Figure 13:**
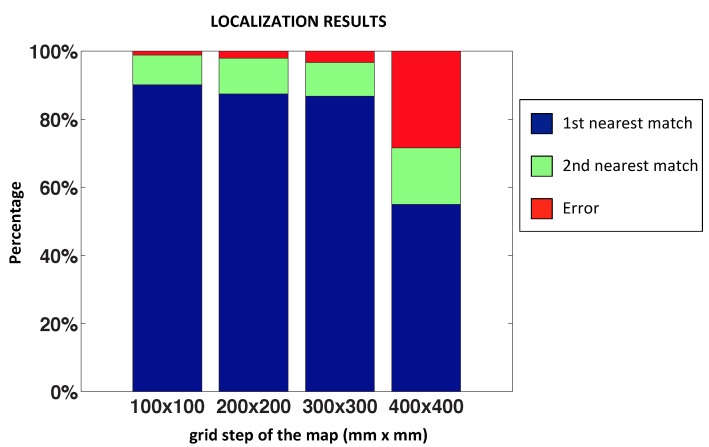
Results of the nearest image experiments: success rate.

Figure 13 shows the final result of this test. We have used 3500 test images captured from different random positions of the environment. In each bar, the blue part represents the proportion of correct localizations; the green part represents that the method has localized the second nearest position (*i.e.,* the position calculated is not the nearest position, but rather the second nearest position of the map); and the red part is the proportion of errors for each map size.

We can observe that the method is working properly with a distance between map positions not exceeding 300 × 300 millimeters, because if this value increases, the POC distance begins to saturate, as stated in Section 2.4.

#### 5.2.2. Second Method: Local Mapping and Localization

In this subsection, the second method is analyzed, and we study the influence of the distance between map positions and the number of nearest neighbors used in the spring algorithm. To do this experiment, we have chosen the same distances between map positions as in the previous subsection: 100, 200, 300 and 400 mm. Then, we will choose the value that provides the best result, and we will then optimize the number of nearest neighbors: we will test grids of 9, 25 and 49 nearest neighbors.

**Figure 14:**
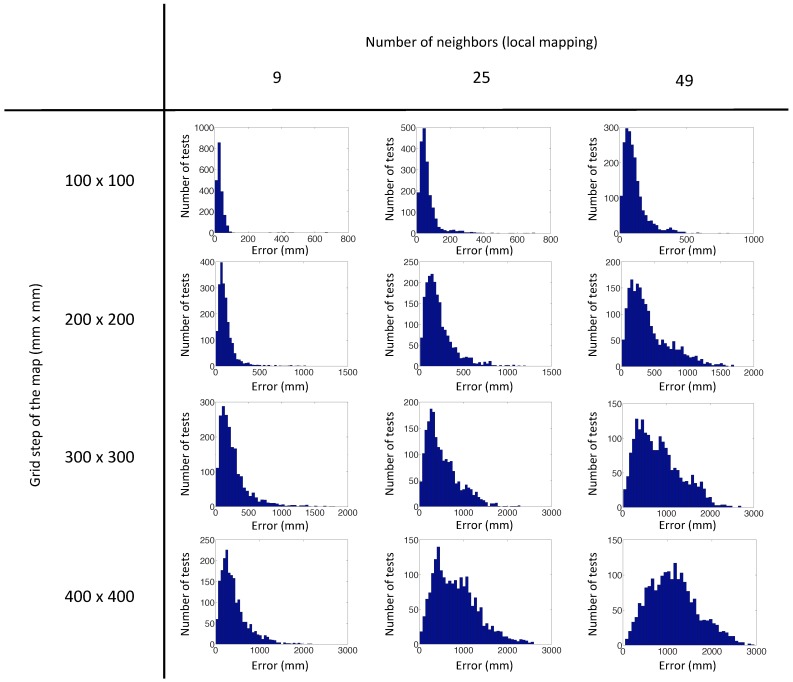
Error distribution of the experiments with our virtual database, using POC distance.

**Figure 15:**
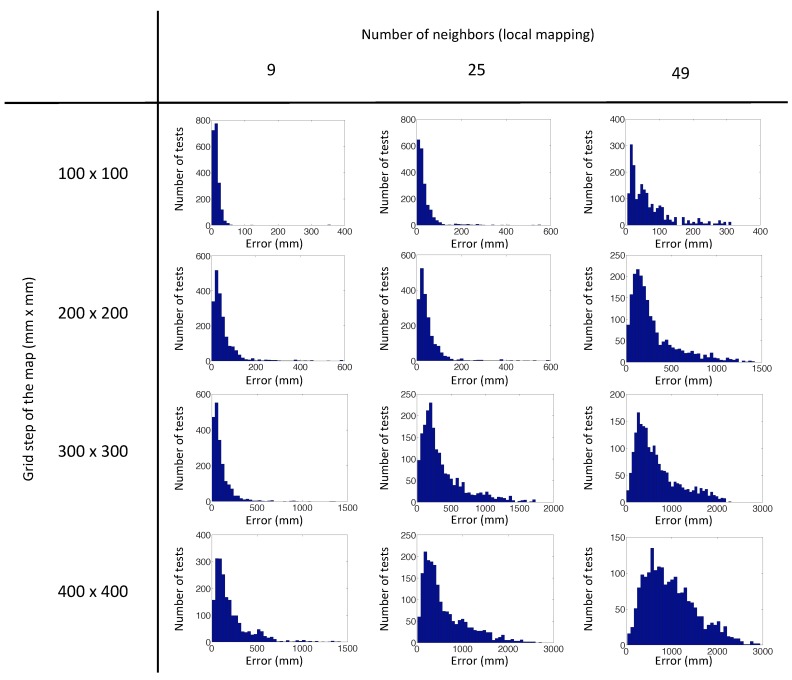
Error distribution of the experiments with our virtual database, using gist distance.

The algorithm has been launched with 500 test images captured from random positions generated with our program, repeating the experiment four times with each test image. Figure 14 shows the error distribution of the experiments with the virtual database using the POC distance, depending on the number of nearest neighbors and the grid step of the map. This error is the localization error of the test image calculated according to Step 5 (Section 4.2). Figure 15 presents the error distribution of the same experiments, but with the gist distance. In this environment, the gist distance presents a lower localization error than the POC distance. The parameters used in the gist descriptors for the experiments are n=2 levels and b=16 horizontal blocks.

**Figure 16:**
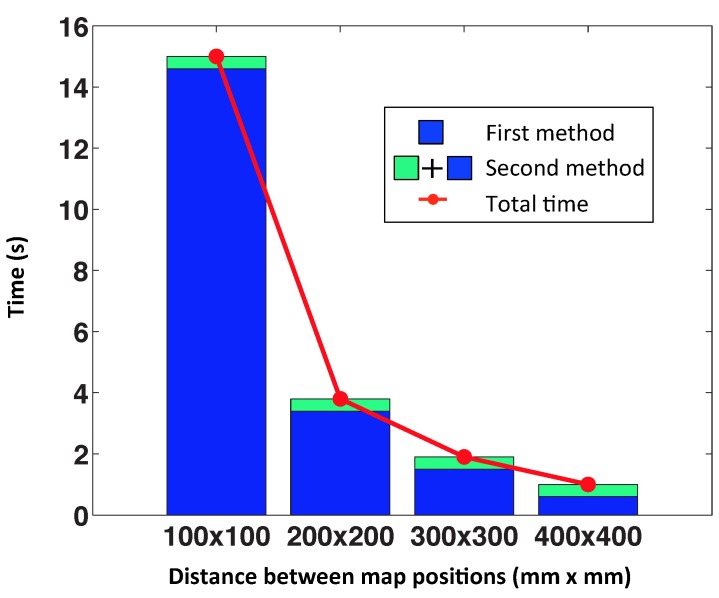
Computational time for each iteration. Using 25 nearest neighbors in the second method.

As we can see in the figures, the best results of the test have been achieved with a grid step equal to 100 mm and 200 mm, but we must also consider the computation time. Figure 16 shows the average computation time per iteration in each case, in the case that we have chosen 25 nearest neighbors. The distance between map positions of 100 mm is not advisable, because the computation time is relatively high. For this reason, we choose the distance of 200 mm to do the following tests, with actual images, because taking into account the error and the computation time, it is the best option. We can observe that the computational time of the second method is the same for all cases, because it does not depend on the size of the map, but rather on the number of nearest neighbors.

### 5.3. Results with Actual Databases

Once we have analyzed our methods with a virtual database, now we will test them with some databases composed of images captured in real working environments under real lighting conditions.

First, we test the first method using a third-party database that has different lighting conditions.

Second, we test the second method with our own databases (two different rooms of our university).

#### 5.3.1. First Method: Detecting the Nearest Image of the Map

To test this method, we have used the Bielefeld database [24]. This database contains several image maps with changes in the position of some objects and in the lighting conditions. The images were captured on a 10 × 17 grid with a 30-cm step. Hence, the area of the capture grid was 2.7 m × 4.8 m, which covered nearly all of the floor’s free space. These image maps are:

- Original: the standard or default condition of the room. Images were collected with both overhead fluorescent light bars on, the curtains and door closed and no extraneous objects present.
- Chairs: three additional office chairs were positioned within the capture grid.
- Arboreal: a tall (3 m) indoor plant was added to the center of the capture grid.
- Twilight: curtains and door open. The image collection lasted from sunset to the evening.
- Doorlit: the light bar near the window was switched off.
- Winlit: the light bar near the door was switched off.

**Figure 17:**
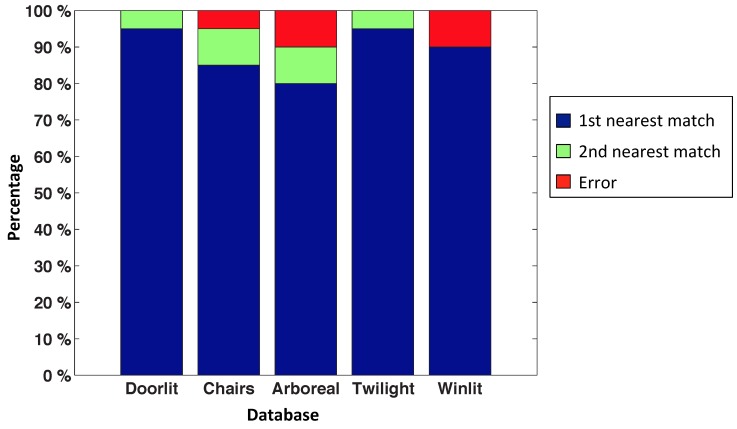
Success rate with the Bielefeld database.

To carry out this first experiment, we have used the “original” set as our map of images and the images in the other sets as test images. Figure 17 shows the success rate of this experiment. We have used 20 random test images of each database to obtain this figure.

This is quite an interesting experiment, as it shows the robustness of the algorithm, even when the test images present changes with respect to the map.

#### 5.3.2. Second Method: Local Mapping and Localization

In this case, we have used our database of images, captured at our university. It covers two different rooms, a laboratory and an office. The images have been captured on a grid with 8 × 8 images with a 20-cm step, in both cases.

**Figure 18:**
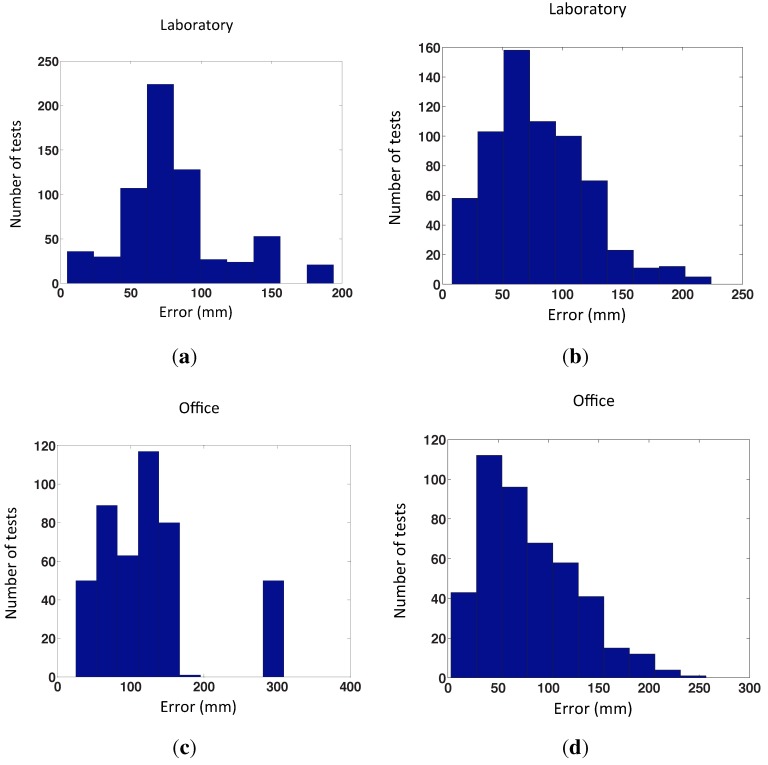
Error distribution of the second method. (**a**,**b**) Laboratory database and gist and POC distances, respectively; (**c**,**d**) Office database and gist and POC distances, respectively. The distance between map positions in the four experiments is 200 × 200 mm.

**Figure 19:**
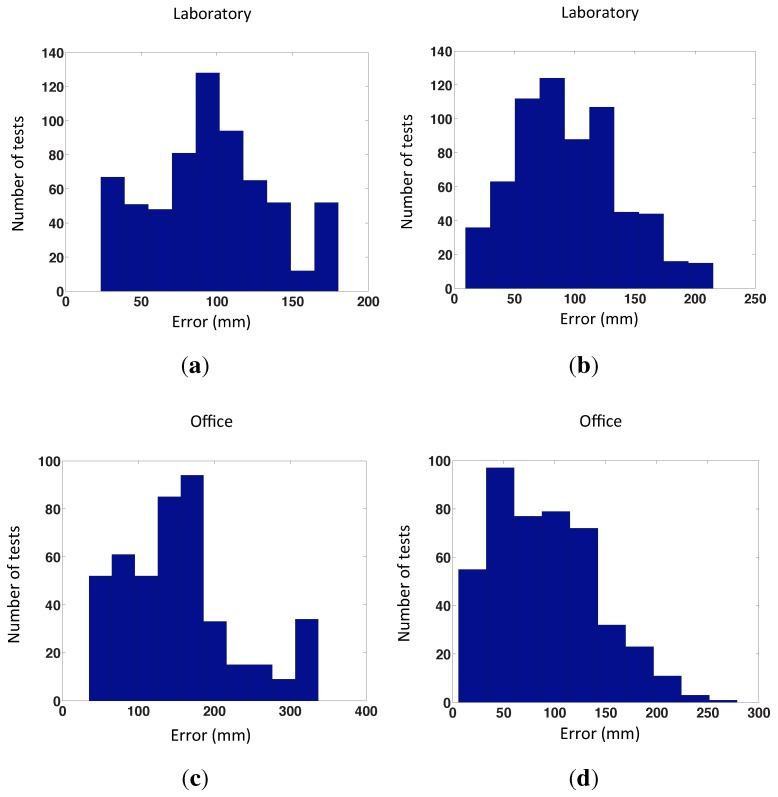
This figure represent the same experiments of Figure 18, adding random occlusions to the test image. (**a**,**b**) Laboratory database and gist and POC distances, respectively; (**c**,**d**) Office database and gist and POC distances, respectively. The distance between map positions in the four experiments is 200 × 200 mm.

**Figure 20:**
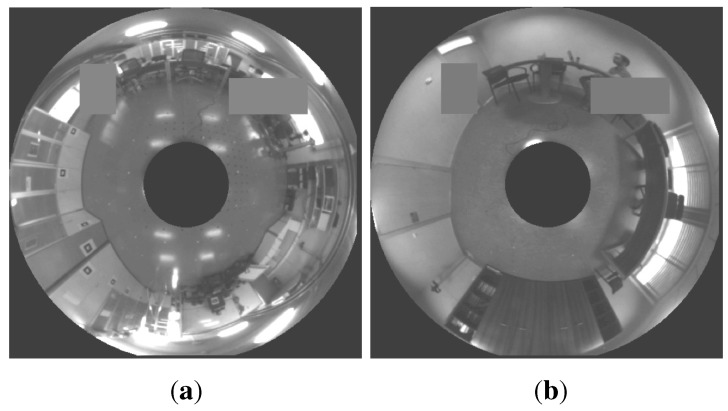
(**a**) Example of an omnidirectional image captured in our laboratory with two occlusions; (**b**) Example of an omnidirectional image captured in our office with two occlusions.

We follow two steps. First, we detect the nearest neighbors by the POC comparison of the Radon transforms of the map images. Second, we create the local map with these Radon transforms using the gist or POC distance. Finally, we localize our robot in this local map. Figure 18 shows the error distribution of the second method using our databases (“laboratory” and “office”) and both distance measures (POC and gist distance). The experiment has been carried out with 13 test images and 50 times per test image in the laboratory and with mine test images and 50 times per test image in the office. Figure 19 shows the error distribution of the second method using our databases, adding occlusions to each test image. Two examples of omnidirectional images with occlusions are shown in Figure 20.

## 6. Discussion

Once we have presented the results, in this section, we provide a discussion of these results with both methods. We have arrived at some conclusions about the advantages and disadvantages of each method with different values of the variable parameters.

The first method (detecting the nearest image of the map) is a very good method if the robot has information of the positions in which the map images were taken. It permits carrying out an absolute localization, because the robot needs no information of its previous location. The results have demonstrated that it is a very reliable method to estimate the nearest position of the map, even when some changes are present (lighting conditions, new objects, *etc.*). As for the values of the parameters, the distance between map positions is the variable parameter in this method. The best choice is 200 × 200 mm, as it offers a good balance between accuracy and computational time.

The second method (local mapping and localization) is a good method if the robot does not know the map positions, because it creates a local map. This method also localizes the nearest images and the test image within this local map. As for the values of the parameters, first, the best choice is a distance between map positions of 200 × 200 mm, as it offers a good balance between accuracy and computational time; second, the best number of nearest neighbors is nine, because it leads to lower error; and finally, the kind of distance (POC or gist distance) should be chosen depending on the characteristics of the environment, because with the virtual database, the gist distance is better, but with the actual database of our university, the POC distance has presented more accurate results.

To finish, a comparative evaluation has been carried out between the proposed descriptor (Radon) and two other classical description methods in a localization task. The objective is to study the performance of the proposed descriptor, both in terms of localization accuracy and computational cost.

The description methods we use to make this comparison are the Fourier signature (FS) [11], a classical method to describe the global appearance of a panoramic scene, and SIFT [6], a typical method to extract and describe local features from a scene. The test consists of determining the nearest image from a set of omnidirectional images, and the database used is the laboratory (from the actual database captured at our university).

**Figure 21:**
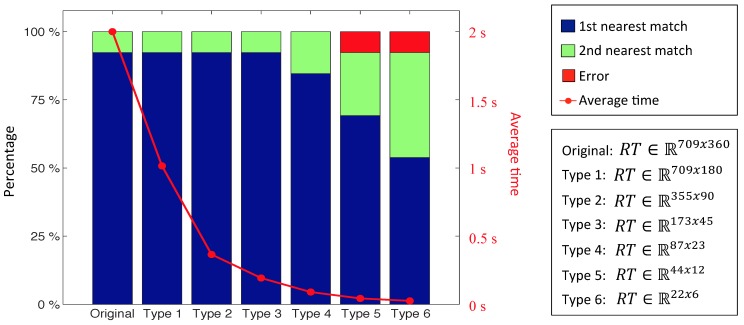
Success rate and average time using different types of Radon transforms with the laboratory database.

First, an experiment has been carried out to optimize the computational cost of the Radon transform (RT) method. With this aim, we repeat the localization experiment several times, using different resolutions, from RT $\in R^{709x360}$ to RT $\in R^{22x6}$. The results (success rate and computational cost) are shown on Figure 21. As shown, RT resolution can be reduced to 173×45 keeping the success rate, which clearly improves the computational cost of the process. This way, we choose this resolution to carry out the comparison.

When using the Fourier signature, each omnidirectional scene is transformed to the panoramic format and described according to [11]. The Euclidean distance is used to compare two descriptors. On the other hand, when using SIFT, the SIFT features of the omnidirectional scenes are extracted, described and compared. The distance between two images $im_{1}$ and $im_{2}$ is defined in Equation (16).

(16) $$dist_{SIFT}\left(im_{1},im_{2}\right)=\frac{1}{\sum_{i=1}^{n}\frac{1}{d_{i}}}$$

where $d_{i}$ is the average distance between each SIFT feature in the first image and its corresponding feature in the second image. *n* is the number of matched SIFT features.

**Figure 22:**
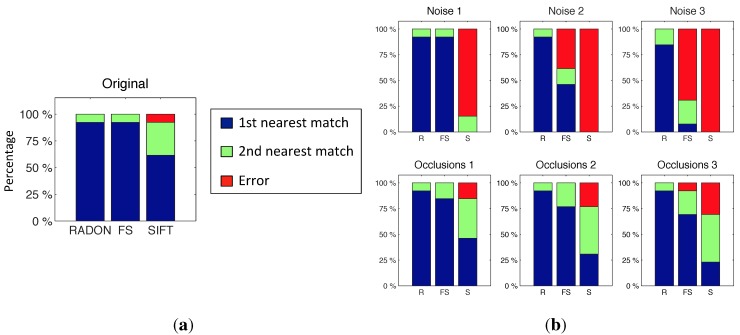
Success rate with the laboratory database. (**a**) Using the original test images; (**b**) Taking into consideration the presence of different levels of noise and occlusions on the test images.

Figure 22a shows the success rate of the comparative experiment using the original test images. Thirteen random test images have been used to obtain this figure. Later, we repeated the experiment taking into consideration the presence of different levels of noise and occlusions on the test images, to test the robustness of each method under these typical situations. The results are shown in Figure 22b. Noises 1, 2 and 3 are random noises with maximum value equal to 20%, 40% and 60% of the maximum intensity. Occlusions 1, 2 and 3 imply that the test image has 10%, 20% and 30% occlusion, respectively.

As for the computational time used to do this experiment, the Radon transform spends 0.17 s; SIFT spends 12 s (using the algorithm of [6]). At last, the Fourier signature method needs 0.06 s.

Taking these results into account, the RT descriptor has been proven to be an effective method. It shows a reasonably good computational cost, and its robustness in the localization process is an important feature. While the FS and SIFT methods have worse behavior as noise and occlusions increase, RT has a good and stable behavior.

**Figure 23:**
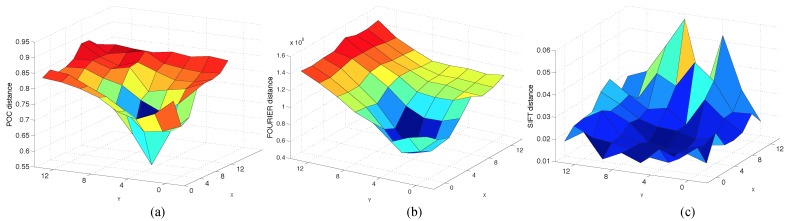
Distance between a sample test image captured at x = 3, y = 3 and all of the map images using (**a**) the Radon descriptor and POC distance; (**b**) Fourier signature (FS) and (**c**) SIFT.

To explore the reason for this behavior, we depict in Figure 23 the distance between one sample test image and each image of the map. The test image is in the position (X,Y)=(3,3). In this figure, the Radon descriptor stands out for its ability to localize the robot sharply since the distance increases quickly around the minimum. To check this performance, we have defined a parameter that measures the sharpness level of the minimum in each case (Equation (17)). The sharper the function, the higher is C.

(17) $$C=\frac{mean(distance)-min(distance)}{max(distance)-min(distance)}\cdot 100\left(\%\right)$$

Using this equation with each descriptor, SIFT, FS and RT and carrying out 13 experiments in each case, the average value of *C* is: 60% using SIFT, 61% using FS and 79% using RT.

## 7. Conclusions

In this paper we have presented two different methods to estimate the position of a robot in an environment in two different situations. In one of them, the robot knows the map positions, and in the other, it only knows the map images. The first method calculates the nearest position of the map with the test image taken by the robot, and the second method creates a local map with the nearest map images and the test image taken by the robot. These methods use omnidirectional images and global appearance descriptors.

We have also developed a method to build global appearance descriptors from omnidirectional images, based on Radon transform.

Both methods are first tested with our virtual database to determinate the best parameters, and they are then tested with actual databases. One of the methods has been analyzed with a database with changes in lighting conditions and adding objects. The other method has been analyzed with two different databases captured at our University.

Furthermore, a comparative analysis between the proposed descriptor and two other classical descriptors has been carried out. This analysis has shown the robustness of our method to solve the localization problem when the test images present noise or occlusions. These are common phenomena in real applications, and the RT method has been proven to be able to cope with them.

The results presented in this paper show the effectiveness of global appearance descriptors of omnidirectional images to localize the robot and to create a map. We are now working to improve these methods, and we are trying to carry out the localization in a map with more degrees of freedom. We are also working to include the color information of the images in the global appearance descriptors.
